# Histone deacetylase inhibitors enhance estrogen receptor beta expression and augment agonist-mediated tumor suppression in glioblastoma

**DOI:** 10.1093/noajnl/vdab099

**Published:** 2021-07-17

**Authors:** Uday P Pratap, Gangadhara R Sareddy, Zexuan Liu, Prabhakar Pitta Venkata, Junhao Liu, Weiwei Tang, Kristin A Altwegg, Behnam Ebrahimi, Xiaonan Li, Rajeshwar R Tekmal, Suryavathi Viswanadhapalli, Stanton McHardy, Andrew J Brenner, Ratna K Vadlamudi

**Affiliations:** 1 Department of Obstetrics and Gynecology, University of Texas Health San Antonio, San Antonio, Texas, USA; 2 Department of Oncology, Xiangya Hospital, Central South University, Changsha, Hunan, PR China; 3 Department of Obstetrics and Gynecology, Affiliated Hospital of Integrated Traditional Chinese and Western Medicine, Nanjing University of Chinese Medicine, Nanjing, PR China; 4 Hematology & Oncology, University of Texas Health San Antonio, San Antonio, Texas, USA; 5 Mays Cancer Center, University of Texas Health San Antonio, San Antonio, Texas, USA; 6 Department of Chemistry, University of Texas San Antonio, San Antonio, Texas, USA

**Keywords:** epigenetic drugs, estrogen receptor β, glioblastoma, panobinostat, romidepsin

## Abstract

**Background:**

Glioblastomas (GBMs) are the most lethal primary brain tumors. Estrogen receptor β (*ESR2/*ERβ) function as a tumor suppressor in GBM, however, ERβ expression is commonly suppressed during glioma progression. In this study, we examined whether drugs that reverse epigenetic modifications will enhance ERβ expression and augment ERβ agonist-mediated tumor suppression.

**Methods:**

We tested the utility of epigenetic drugs which act as an inhibitor of histone deacetylases (HDACs), histone methylases, and BET enzymes. Mechanistic studies utilized RT-qPCR, chromatin immunoprecipitation (ChIP), and western blotting. Cell viability, apoptosis, colony formation, and invasion were measured using in vitro assays. An orthotopic GBM model was used to test the efficacy of in vivo.

**Results:**

Of all inhibitors tested, HDACi (panobinostat and romidepsin) showed the potential to increase the expression of ERβ in GBM cells. Treatment with HDACi uniquely upregulated ERβ isoform 1 expression that functions as a tumor suppressor but not ERβ isoform 5 that drives oncogenic functions. Further, combination therapy of HDACi with the ERβ agonist, LY500307, potently reduced cell viability, invasion, colony formation, and enhanced apoptosis. Mechanistic studies showed that HDACi induced ERβ is functional, as it enhanced ERβ reporter activities and ERβ target genes expression. ChIP analysis confirmed alterations in the histone acetylation at the ERβ and its target gene promoters. In orthotopic GBM model, combination therapy of panobinostat and LY500307 enhanced survival of tumor-bearing mice.

**Conclusions:**

Our results suggest that the combination therapy of HDACi and LY500307 provides therapeutic utility in overcoming the suppression of ERβ expression that commonly occurs in GBM progression.

**Key Points**

- HDACi such as panobinostat and romidepsin can enhance the expression of tumor suppressor ERβ in GBM.
- Combination therapy of HDACi and ERβ agonist provides therapeutic utility in GBM.

**Importance of the Study**

Estrogen receptor β (ERβ) function as a tumor suppressor in glioblastoma (GBM), however, ERβ expression is commonly downregulated during glioma progression. Our study shows that inhibitors of histone deacetylases (HDACi) such as panobinostat and romidepsin enhance the expression of ERβ and its target genes by altering histone acetyl marks at promoter regions. More importantly, combination therapy of HDACi with the ERβ agonist LY500307 potently reduced cell viability, invasion, colony formation, and enhanced apoptosis. In orthotopic GBM model, combination therapy of panobinostat and LY500307 enhanced survival of tumor-bearing mice. Our studies implicate that upregulation of ERβ expression/functions by HDACi along with ERβ agonist is an attractive therapy for GBM.

Glioblastoma (GBM) is one of the most commonly diagnosed and aggressive forms of primary malignant brain tumors in adults.^1,2^ GBM is considered as one of the most deadly neoplasms with the worst 5-year overall survival rates among all human cancers.^3^ Specifically, the prognosis for GBM is 65% survival up to 1-year with a 5-year survival of 12%.^4^ GBMs affect 13 000 patients per year in the United States.^2,5^ Current standard of care for GBM consists of surgically excising the tumor, in conjunction with external beam radiation therapy (XRT), and adjuvant chemotherapy with temozolomide.^4,6^ However, the development of resistance to XRT and chemotherapy is a major clinical problem.^7,8^ Therefore, rationally designed strategies that will improve the response to GBM treatments and extend survival are urgently needed.

The biological effects of estrogen (17β-estradiol, E2) are mediated through 2 distinct estrogen receptors: estrogen receptor α (ERα/*ESR1*) and estrogen receptor β (ERβ/*ESR2*). These ER subtypes have unique biological functions. For example, ERβ exhibits antitumor activity, unlike ERα.^9^ Several studies have shown that overexpression of ERβ reduces cell proliferation, whereas knockdown of ERβ enhances cell proliferation in cancer cells.^10,11^ As transcription factors, ERα and ERβ share many target genes; however, ERβ activates a unique set of genes^12,13^ via its direct DNA binding or its interactions with other transcription factors.^12,14^ GBM cells uniquely express ERβ,^15^ further, recent evidence indicates that the expression of ERβ is significantly reduced during GBM progression. Therefore, understanding the molecular mechanisms that contribute to downregulation of ERβ and identifying novel targets that drive ERβ expression are necessary to enhance ERβ-mediated tumor suppression in GBM.

Histone post-translational modifications contribute to tumor progression and enzymes that regulate these modifications such as acetyltransferases and deacetylases are suggested to play a vital role in GBM pathogenesis.^16^ Histone deacetylase inhibitors (HDACi) have been identified as novel agents and are being explored as potential therapeutics against several cancers, including GBM.^17^ HDACi function as anticancer agents via multiple mechanisms, by upregulating expression of tumor suppressor genes, inhibiting oncogenes, and by modulating the tumor microenvironment.^18^ HDACi such as vorinostat, romidepsin, belinostat, and panobinostat are approved by the FDA for the treatment of hematological malignancies. However, the molecular mechanism(s) by which HDACi promote GBM suppression is elusive.

In this study, we examined the role of HDACi in modulating the expression of the tumor suppressor gene ERβ. Using established and primary GBM cells, we showed that HDACi such as panobinostat and romidepsin enhance the expression of ERβ. Chromatin immunoprecipitation (ChIP) assays demonstrated that HDACi enhanced the acetylation of the ERβ and its genes promotors. Reporter gene, and RT-qPCR assays confirmed that HDACi promoted the activation of ERβ signaling. HDACi enhanced ERβ agonist-mediated suppression of cell viability, survival, and invasiveness of GBM cells and enhanced apoptosis. Importantly, HDACi and ERβ agonist LY500307 combination therapy enhanced overall mouse survival in orthotopic GBM models.

## Materials and Methods

### Cell Lines and Reagents

GBM cell lines U87 and U251 were purchased from the American Type Culture Collection (ATCC) and were maintained as per ATCC guidelines. GBM cells were maintained in Dulbecco’s modified Eagle’s medium (DMEM) supplemented with 10% fetal bovine serum (Sigma Chemical Co.). For all the biological and reporter gene assays, cells were cultured in phenol red-free media with 5% dextran-coated charcoal stripped (DCC) serum for 48 h before initiating actual treatments to avoid effects of endogenous estrogen. Neurobasal medium and B27 serum-free supplement were purchased from Invitrogen. ERβ antibody (Cat # GTX70174, WB-1:200, IHC-1:100, ICC 1:100) was purchased from GeneTex and p21 Waf1/Cip1 (Cat # 2947S, WB-1:1000) antibody was purchased from Cell Signaling Technology, Ki67 antibody (Cat # ab16667, IHC-1:50) was purchased from Abcam, β-actin (Sigma, Cat # A2066, WB-1:1000) and all secondary antibodies were purchased from Cell Signaling and Millipore Sigma Chemical Co. ERβ agonist LY500307 was purchased from Cayman Chemical. Cell identity was confirmed with Short Tandem Repeat polymorphism analysis. CellTiter-Glo Luminescent Cell Viability Assay kit and Dual Luciferase Assay system were obtained from Promega. HDACi (panobinostat and romidepsin) were purchased from MedChem Express. ERβ-FLAG overexpression model cells were generated by infecting them with pLenti6/V5-D-FLAG ERβ and empty control vectors and stable clones were selected with blasticidin (5 µg/mL). ERβ-knockout cells were generated as described previously.^19^ Annexin V/PI kit was purchased from BioLegend.

### Patient-Derived Primary GBM Cells

Primary GBM cells were isolated from discarded specimens obtained from GBM patients undergoing surgery at UT Health San Antonio under an IRB approved biorepository. All samples were deidentified by the biorepository before provision. Patients provided informed consent for surgery and use of their tissues for research. Primary GBM lines, GSC-101310, GSC-111010, and GSC-012015, were cultured as neurospheres in neurobasal medium supplemented with B27 serum-free supplement, epidermal growth factor (EGF) (20 ng/mL), basic fibroblast growth factor (bFGF) (20 ng/mL), leukemia inhibitory factor (LIF) (10 ng/mL), and heparin (5 µg/mL) as described previously.^20,21^

### Cell Viability and Clonogenic Assays

The cell viability rates of GBM cells treated with HDACi and LY500307 were measured by using MTT and Cell Titer-Glo Luminescent Cell Viability Assays. U251 and U87 cells (5 × 10^3^ cells/well) were seeded in a 96-well plate phenol red-free DMEM media with 5% DCC serum and treated with HDACi (panobinostat or romidepsin) or LY500307 alone or in combination for 6 days and the cell viability rates were determined using MTT assay. Primary GBM cells (5 × 10^3^ cells/well) were seeded in 96-well, flat, clear-bottom, opaque-wall microplates and treated with HDACi or LY500307 alone or in combination for 6 days. Total ATP content as an estimate of total number of viable cells was measured using CellTitier-Glo assay on automatic Promega Luminometer according to the manufacturer’s instructions. For the clonogenic assays, U251 and U87 cells were seeded in 6-well plates, treated with HDACi or LY500307 or combination for 5 days and after 14 days cells were then fixed with ice cold methanol followed by staining with 0.5% crystal violet solution. Colonies were counted and used in the analysis.

### Cell Invasion and Annexin V Assays

The invasive ability of GBM cells that were treated with either vehicle, HDACi or LY500307, or combination was determined using Corning BioCoat Growth Factor Reduced Matrigel Invasion Chamber assay according to the manufacturer’s instructions. Briefly, 2 × 10^4^ cells were seeded in the upper chamber in serum-free medium with 10% FBS containing media as a chemoattractant in bottom well. After 12 h, the invaded cells on the bottom side of the membrane were fixed in methanol and stained with 0.5% crystal violet. The number of invaded cells in 5 random fields was counted and used for quantitative analysis. The Annexin V assay was performed as described previously.^21^ Briefly, GBM cells were treated with either vehicle or HDACi or LY500307 or combination for 48 h. Cells were then harvested and 100 µL of the cell suspension was incubated with Annexin V FITC and propidium iodide for 15 min at room temperature in the dark. Annexin V binding buffer (400 µL) was then added to each sample and stained cells were analyzed using flow cytometry.

### Western Blotting

For western blotting analyses, whole cell lysates were prepared from GBM cells using RIPA buffer containing protease and phosphatase inhibitors (Sigma-Aldrich). Total proteins were run on SDS–PAGE gels followed by transfer onto nitrocellulose membranes. Primary antibody incubation was carried out at 4°C for overnight followed by incubation with secondary antibodies for 1 h at room temperature. Blots were developed using the ECL kit (Thermo Fisher Scientific).

### Reporter Gene Assays

Reporter gene assays were performed as described earlier.^22^ U251 cells expressing ERβ were stably transfected with an Estrogen Response Element (ERE) reporter or U87 cells expressing ERβ were transiently transfected with ERE reporter. Cells were plated in 24 well plates in phenol red-free DMEM media with 5% DCC serum. ERE reporter (3X ERE TATA luc) with 3 copies of ERE sequence (GGTCA CAG TGACC) was purchased from Addgene (plasmid # 11354). U87 cells expressing ERβ were transiently transfected with 250 ng of ERE/LUC reporter plasmid using Turbofect transfection reagent (Thermo Scientific, MA). Renilla luciferase plasmid (20 ng) was cotransfected and used for data normalization. Cells were treated with HDACi or LY500307 or combination for 24 h. Cells were then lysed in Luciferase Lysis Buffer, and the luciferase activity was measured by using the Dual Luciferase Assay system (Promega) with a luminometer.

### ChIP Assay

ChIP assay was performed using Pierce Magnetic ChIP Kit (Thermo Fisher Scientific) as per the manufacturer’s protocol. Briefly, 4 × 10^6^ cells were crosslinked using 1% formaldehyde for 10 min, lysed and digested with micrococcal nuclease. Chromatin was immunoprecipitated using 3.0 µg of H3K9-Ac antibody (07-352, Millipore Sigma) or 3.0 µg of isotype-specific IgG antibody. Recovered ChIP DNA was dissolved in 50 µL TE buffer and used for PCR amplification using the ERβ 0N promoter, forward primer sequence: 5′-CCACTATCCTTGTGGGTGGA-3′, reverse primer sequence: 5′-CAGCAGCTGGAGAAACTGAA-3′; MDA7 promoter, forward primer sequence: 5′-CCCCATCGCTGTATTGTCCT-3′, reverse primer sequence: 5′-GGAAAAAGAGGGAGGTGGAGA-3′; NKG2E promoter, forward primer sequence: 5′-AGCCACCCAAAGTCTCCTAT-3′, reverse primer sequence: 5′-TTCAGTGGAGAGGTCAGGTT-3′.

### RT-qPCR

ERβ target genes were validated using quantitative real-time PCR (qRT-PCR) using gene-specific primers obtained from Harvard Primer Bank (http://pga.mgh.harvard.edu/primerbank). Data were normalized to GAPDH or β-actin and the difference in fold change was calculated using delta-delta-CT method. Primers sequences used for ERβ isoforms 1 and 5 are as follows: ERβ1-forward: 5′-GTCAGGCATGCGAGTAACAA-3′; ERβ1-reverse: 5′-GGGAGCCCTCTTTGCTTTTA-3′; ERβ5-forward: 5′-GATGCTTTGGTTTGGGTGAT-3′; ERβ5-reverse: 5′-CCTCCGTGGAGCACATAATC-3′; primers sequences used for ERβ target genes are follows: MDA7-forward: 5′-CTTTGTTCTCATCGTGTCACAAC-3′; MDA7-reverse: 5′-TCCAACTGTTTGAATGCTCTCC-3′; NKG2E-forward: 5′-GCCAGCATTTTACCTTCCTCAT-3′; NKG2E-reverse: 5′-AACATGATGAAACCCCGTCTAA-3′; HAVCR2- forward: 5′-GAAGAAGAAGCAGTGACGGG-3′; HAVCR2- reverse: 5′-TGTCAGAATTGTGCTAGGCG-3′; PLA2G4D- forward: 5′-AGCCCCGGATCTGCTTTCT-3′; PLA2G4D-reverse: 5′-GGTGAGGTCATACCAGGCATC-3′; P21- forward: 5′-CTGGAGACTCTCAGGGTCGAAA-3; P21-reverse: 5′-GATTAGGGCTTCCTCTTGGAGAA-3′; GADD45B-forward: 5′-TACGAGTCGGCCAAGTTGATG-3′; GADD45B-reverse: 5′-GGATGAGCGTGAAGTGGATTT-3′; GAPDH-forward: 5′-TGTTACCAACTGGGACGACA-3′; GAPDH-reverse: 5′-GGGGTGTTGAAGGTCTCAAA-3; Actin-forward: 5′-GTGGGCATGGGTCAGAAG-3′; Actin-reverse: 5′-TCCATCACGATGCCAGTG-3′. RT-qPCR was performed using SYBR Green (Thermo Fisher Scientific) on an Illumina Real-Time PCR system.

### Immunocytochemistry

U251-WT and U251-ERβ-KO cells were seeded in poly-d-lysine coated chamber slides and after 48 h cells were fixed with 4% paraformaldehyde. Cells were subjected to permeabilization with 0.5% Triton X-100 followed by 2 times phosphate-buffered saline wash for 5 min each. Cells were then blocked in 5% bovine serum albumin followed by incubation with ERβ primary antibody overnight and horseradish peroxidase-conjugated secondary antibody for 1 h at room temperature. Immunoreactivity was detected by using the diaminobenzidine (DAB) substrate and the cells were counterstained with hematoxylin (Vector Lab, Inc.).

### In Vivo Orthotopic Xenograft Model and Immunohistochemistry

All animal experiments were conducted after obtaining UT Health San Antonio IACUC approval. Male 8–10 weeks old SCID mice were purchased from Charles River. U251 cells stably labeled with GFP-Luciferase reporter (1 × 10^6^ cells) were orthotopically injected into the right cerebrum of the mouse using an established protocol.^21^ Tumor progression was monitored weekly using the Xenogen in vivo imaging system. Mouse survival (*n* = 6/group) was determined using Kaplan–Meier survival curves and log-rank test using GraphPad Prism 8 software (GraphPad Software). Immunohistochemical studies were performed as described previously.^23^ Briefly, tumor tissue sections were incubated overnight with Ki67 and ERβ antibodies followed by secondary antibody incubation for 30 min at room temperature. Immunoreactivity was detected by using the DAB substrate and counterstained with hematoxylin (Vector Lab, Inc.). Percentage of Ki67-positive proliferating cells and staining of ERβ was calculated from 5 randomly selected microscopic fields.

### Statistical Analysis

Statistical differences between the groups were analyzed with Student’s *t*-test or analysis of variance as appropriate using GraphPad Prism 8 software. All the data represented in the graphs are shown as means ± SE. A value of *P* < .05 was considered as statistically significant.

### Data Accessibility

All the data generated and/or analyzed during the current study are included in this article and are available from the corresponding author on reasonable request.

### Ethics Statement

All animal experiments were performed in accordance with IACUC standards and by using approved protocols at UT Health San Antonio.

## Results

### HDACi Enhances Expression of ERβ in GBM Cells

**Figure 1.**
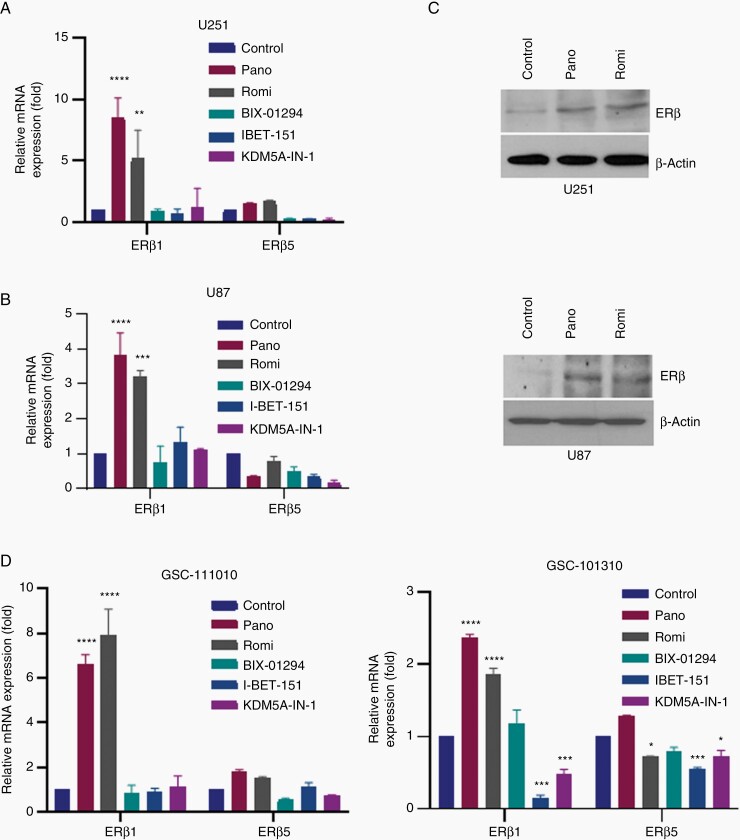
HDACi enhance expression of ERβ. (A) U251 cells were treated with histone modifying enzymes for 24 h (Pano 12.5 nM, Romi 6.25 nM, BIX-01294 1000 nM, I-BET-151 1000 nM, KDM5A-IN-1 2000 nM) and the expression of ERβ isoforms 1 and 5 was determined by isoform-specific RT-qPCR primers. (B) U87 ells were treated with indicated inhibitors of histone modifying enzymes for 24 h (Pano 50 nM, Romi 3.1 nM, BIX-01294 750 nM, I-BET-151 1000 nM, KDM5A-IN-1 2000 nM) and the expression of ERβ isoforms 1 and 5 was determined by isoform-specific RT-qPCR primers. (C) U251 cells were treated panobinostat (12.5 nM) and romidepsin (6.25 nM) for 24 h, and the expression of ERβ was measured using western blotting (upper panel). U87 cells were treated panobinostat (50 nM) and romidepsin (6.25 nM) for 24 h, and the expression of ERβ was measured using western blotting (lower panel). (D) Primary GBM cells GSC-111010 (Pano100 nM, Romi 25 nM, BIX-01294 700 nM, I-BET-151 2000 nM, KDM5A-IN-1 2000 nM) and GSC-101310 (Pano 50 nM, Romi 6.25 nM, BIX-01294 1000 nM, I-BET-151 1000 nM, KDM5A-IN-1 2000 nM) were treated with indicated concentrations of inhibitors of histone modifying enzymes for 24 h, the status of ERβ isoforms 1 and 5 was determined using isoform-specific RT-qPCR primers. Data are representative of 3 independent experiments (*n* = 3). Data are represented as mean ± SEM. *P < .05; **P < .01; ***P < .001; ****P < .0001. Statistical differences were examined using 2-way ANOVA. ANOVA, analysis of variance; ERβ, estrogen receptor β; GBM, glioblastoma; HDACi, histone deacetylase inhibitors.

To examine whether inhibition of histone modifying enzymes upregulates expression of ERβ, we used 5 inhibitors of histone modifying enzymes. These include histone methyltransferase G9a inhibitor (BIX-01294), BET inhibitor for BRD2, BRD3, and BRD4 proteins (I-BET151), histone lysine demethylases 5A inhibitor (KDM5A-IN-1), and 2 different HDAC inhibitors (panobinostat and romidepsin). GBM model cells (U251 and U87) were treated with indicated inhibitors and after 24 h, ERβ mRNA was measured. GBM cells predominantly express 2 different ERβ isoforms (ERβ1 and ERβ5).^19^ Since ERβ1 and ERβ5 isoforms are regulated by distinct promoters 0N and 0K, respectively, and possess contrasting functions in GBM, we examined whether epigenetic enzyme inhibitors upregulate the expression of both isoforms of ERβ using RT-qPCR. Of the 5 inhibitors tested, only HDAC inhibitors (panobinostat and romidepsin) increased the levels of ERβ isoform 1 but not ERβ isoform 5 (Figure 1A and B). We also confirmed that these inhibitors increase expression of ERβ at the protein level using western blot analysis (Figure 1C). We further confirmed these findings using 2 additional primary GBM cells. RT-qPCR analyses showed that both panobinostat and romidepsin uniquely upregulate expression of ERβ1 with no or limited alteration in the levels of ERβ5 in primary GBM cells (Figure 1D). Collectively, these results suggest that HDACi such as panobinostat and romidepsin have the potential to uniquely upregulate expression of ERβ isoform 1 which functions as a tumor suppressor.

### HDACi Promotes Acetylation Changes at the ERβ (ERβ1) Promoter and Contributes to Activation of ERβ Target Genes

**Figure 2.**
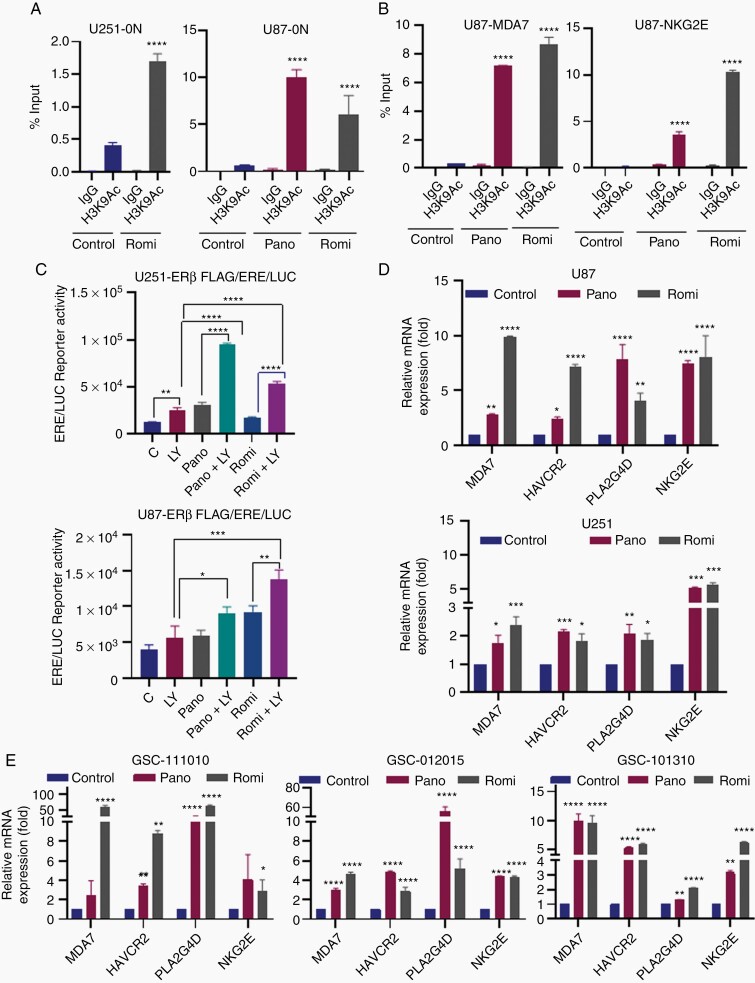
HDACi enhance expression of ERβ target genes. GBM cells (U251 and U87) were treated with vehicle or panobinostat (12.5 nM for U251, 50 nM for U87) or romidepsin (6.25 nM) for 48 h and the status of activation mark H3K9-Ac at the 0N promoter of ERβ (A) and ERβ target genes (B) were analyzed by ChIP. (C) U251-ERβ cells stably expressing ERE-luc reporter and U87-ERβ cells transiently transfected with ERE-luc reporter were treated with LY500307 (10 nm) or panobinostat (6.25 nm for U251, 50 nm for U87) or romidepsin (3.1 nm for U251, 6.25 nM for U87) and the reporter activity was measured after 24 h. U251 (Pano 12.5 nM, Romi 6.25 nM) and U87 (Pano 50 nM, Romi 6.1 nM) (D) and primary GSC-111010 (Pano 100 nM, Romi 25 nM), GSC-012015 (Pano 50 nM, Romi 20 nM), and GSC-101310 (Pano 50 nM, Romi 6.25 nM) (E) cells were treated with panobinostat and romidepsin for 24 h, and the status of ERβ target genes was determined using RT-qPCR. Data are representative of 3 independent experiments (*n* = 3). Data are represented as mean ± SEM. *P < .05; **P < .01; ***P < .001; ****P < .0001. Statistical differences were examined using Student’s *t*-test way or 2-way ANOVA. ANOVA, analysis of variance; ChIP, chromatin immunoprecipitation; ERβ, estrogen receptor β; GBM, glioblastoma; HDACi, histone deacetylase inhibitors.

To examine the mechanism by which HDACi promoted increased expression of ERβ, we examined the acetylation changes at the 0N promoter which regulates expression of the isoform ERβ1. U251 and U87 cells were treated with vehicle or panobinostat or romidepsin for 48 h and the status of activation mark H3K9-Ac at the 0N promoter of ERβ and ERβ target genes (MDA7 and NKG2E) promoters was analyzed by ChIP. Results from ChIP assays showed that HDACi treatment promoted the enrichment of active histone mark H3K9-Ac at ERβ-0N as well as MDA7 and NKG2E promotors (Figure 2A and B). To confirm the functionality of HDACi induced ERβ, we have utilized ERE reporter assays. In ERβ-ERE reporter assays using both U251 and U87 cells, panobinostat and romidepsin treatment significantly enhanced ERE reporter activity which was further enhanced by ERβ agonist treatment, confirming the functionality of HDACi induced ERβ (Figure 2C). Accordingly, HDACi significantly induced the expression of ERβ target genes in both established and primary GBM cells (Figure 2D and E).

### HDACi Enhances the Tumor Suppressive Functions of ERβ Agonist in Reducing Cell Viability and Cell Survival

**Figure 3.**
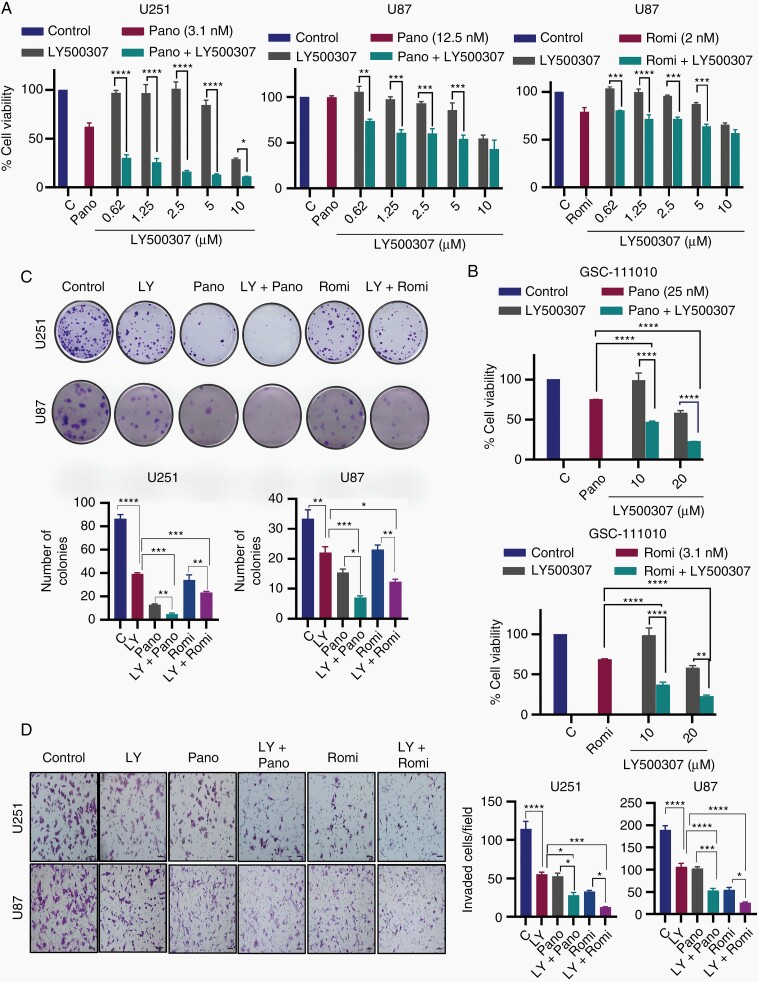
HDACi enhance ERβ agonist LY500307 ability to reduce cell viability, colony formation, and invasion of GBM cells. (A) U251 and U87 cells were treated with indicated concentrations of LY500307 for 6 days in the presence or absence of panobinostat and romidepsin, and the cell viability was measured by MTT assay. (B) Primary GBM cells (GSC-111010) were treated with indicated concentrations of LY500307 for 6 days in the presence or absence of panobinostat or romidepsin, and the cell viability was measured by cell titer glow assay. (C) Effect of HDACi and LY500307 combination therapy on the cell survival of U251 (Pano 3.1 nM, Romi 1 nM, LY500307 5 µM) and U87 (Pano 12.5 nM, Romi 1 nM, LY500307 5 µM) cells was measured using colony formation assays (*n* = 3). Representative images of colonies are shown. Quantitation of colonies is presented. (D) Effect of HDACi and LY500307 combination therapy on cell invasion of U251 (Pano 12.5 nM, Romi 6.25 nM, LY500307 5 µM) and U87 (Pano 25 nM, Romi 6.25 nM, LY500307 5 µM) cells was determined using Matrigel Invasion Chamber assays. Representative images of invaded cells are shown and the number of invaded cells in 5 random fields was quantitated. Data are representative of 3 independent experiments (*n* = 3). Data are represented as mean ± SEM. *P < .05; **P < .01; ***P < .001; ****P < .0001. Statistical differences were examined using 1- and 2-way ANOVA. ANOVA, analysis of variance; ERβ, estrogen receptor β; GBM, glioblastoma; HDACi, histone deacetylase inhibitors.

We next examined the effect of HDACi in the presence of increasing concentrations of ERβ agonist LY500307 on the cell viability and survival of GBM cells. Cell viability assays demonstrated that HDACi treatment increases the efficacy of LY500307 in reducing the cell viability of U251 and U87 and GSC-111010 primary GBM cells (Figure 3A and B). HDACi treatment also increased the efficacy of LY500307 in reducing survival of GBM cells (Figure 3C). Since GBM is highly invasive, which contributes to poor prognosis of patients with GBM, we performed invasion assays. In Matrigel Invasion Chamber assays, HDACi treatment significantly enhanced LY500307-mediated reduction of the invasiveness in GBM cells (Figure 3D). Collectively, these results provide evidence that HDACi has the potential to enhance ERβ agonist-mediated tumor suppressor functions in GBM cells.

### HDACi Enhances ERβ Agonist-Mediated Apoptosis of GBM Cells

**Figure 4.**
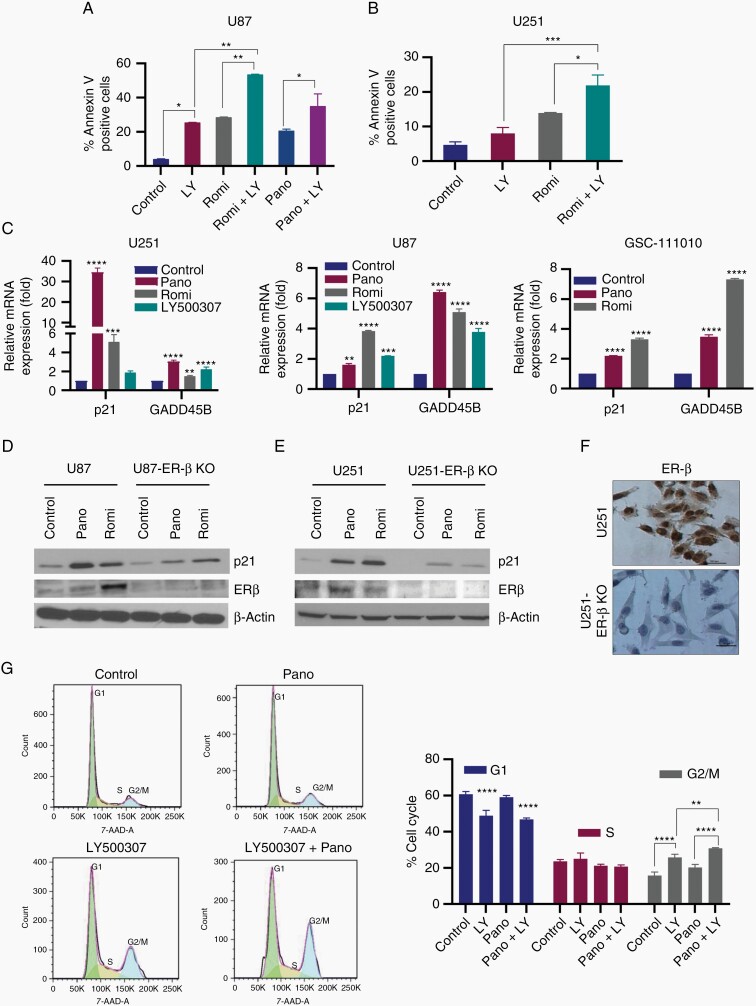
HDACi enhance ERβ agonist LY500307-mediated apoptosis. GBM cells U87 (A) and U251 (B) were treated with vehicle, romidepsin (6.25 nM) or panobinostat (50 nM for U87, 12.5 nM for U251) or LY500307 (10 µM) or in combination for 48 h and the apoptosis was determined using Annexin V-PI staining (*n* = 2). (C) U251 (Pano 12.5 nM, Romi 6.25 nM, LY500307 5 µM), U87 (Pano 50 nM, Romi 6.25 nM, LY500307 5 µM), and GSC-111010 (Pano 100 nM, Romi 25 nM) cells were treated with romidepsin or panobinostat or LY500307 and the status of ERβ target genes involved in cell cycle arrest (p21) and apoptosis (GADD45B) was analyzed by qRT-PCR analysis. (D) U87 and U87-ERβ-KO cells were treated with panobinostat (50 nM) or romidepsin (6.25 nM) for 24 h and the expression of p21 and confirmation of ERβ-knockdown was determined using western blotting. (E) U251, U251-ERβ-KO cells were treated with panobinostat (12.5 nM) or romidepsin (6.25 nM) for 24 h and the expression of p21 and confirmation of ERβ-knockdown was measured using western blotting. (F) ERβ-knockdown in U251-ERβ-KO cells was confirmed using immunocytochemistry. (G) U251 cells were treated with either vehicle or LY500307 or panobinostat or combination for 48 h, and the cells were fixed in 70% ethanol and subjected to PI staining for 20 min. Cell cycle distribution was analyzed using flow cytometry. Data are representative of 3 independent experiments (*n* = 3). Data are represented as mean ± SEM. *P < .05; **P < .01; ***P < .001; ****P < .0001. Statistical differences were examined using 1- and 2-way ANOVA. ANOVA, analysis of variance; ERβ, estrogen receptor β; GBM, glioblastoma; HDACi, histone deacetylase inhibitors; PI, propidium iodide.

Published studies showed that both HDACi^24^ and ERβ agonists^22,23^ promote apoptosis. Since our results indicated that HDACi induce expression of ERβ, we examined whether HDACi enhance ERβ agonist-mediated apoptosis. U251 and U87 cells were treated with LY500307 or HDACi (romidepsin or panobinostat) alone or in combination and apoptosis was analyzed using Annexin V assay. Results showed that HDACi and LY500307 treatment significantly increased the apoptosis in U87 and U251 GBM cells, however combination treatment is highly efficacious in inducing the apoptosis compared to single treatment (Figure 4A and B). Mechanistic studies further confirmed increased expression of ERβ target genes that promote cell cycle arrest and apoptosis including P21 and GADD45B following HDACi and LY500307 treatment (Figure 4C). To examine whether ERβ play a role in HDACi induced p21 expression, we used CRISPR/Cas9 generated U251-ERβ-KO and U87-ERβ-KO cells. Results showed that HDACi treatment induced the expression of p21 in U251 and U87 cells and induction was substantially attenuated in ERβ-KO cells (Figure 4D and E). We also confirmed ERβ-KO in U251 cells using immunocytochemistry (Figure 4F). Further, cell cycle analyses showed that combination treatment of HDACi and LY500307 significantly increased the G2/M cell cycle arrest compared to individual treatment (Figure 4G). Collectively, these results suggest that HDACi have potential to enhance ERβ agonist-mediated apoptosis and cell cycle arrest.

### HDACi and ERβ Agonist Combination Treatment Improves the Survival of Mice Bearing GBM Xenografts

**Figure 5.**
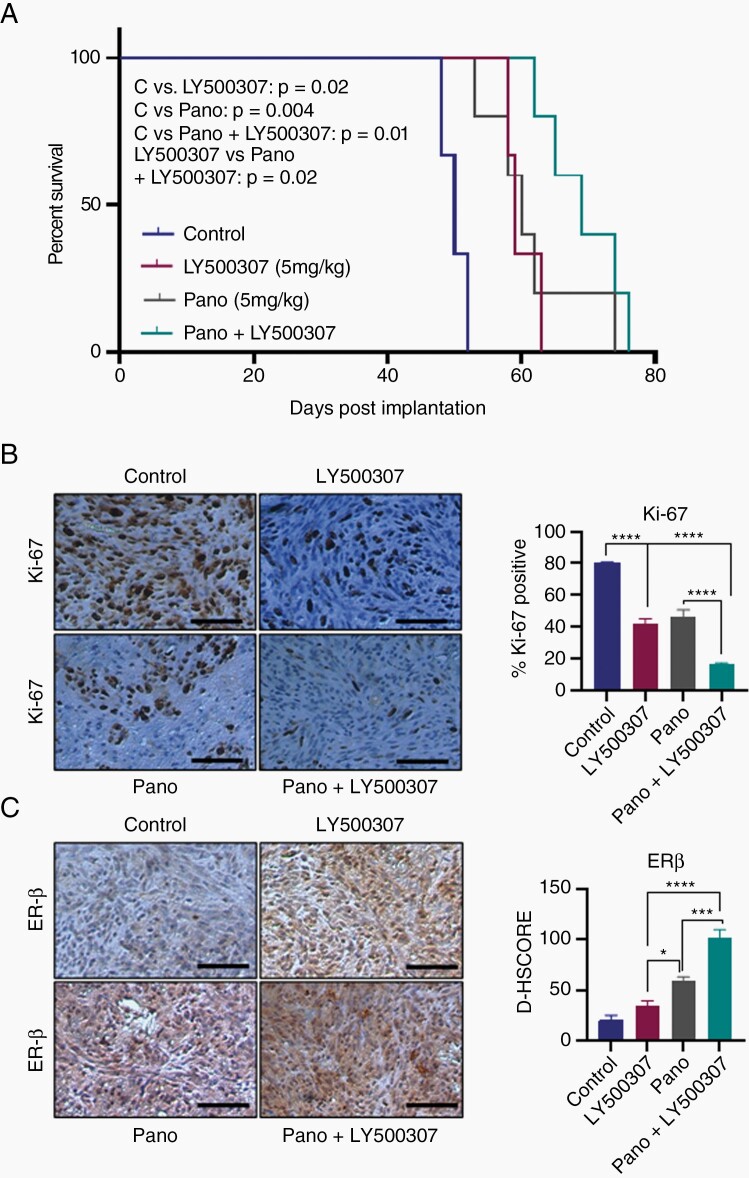
HDACi and LY500307 combination treatment improves the mice survival. (A) SCID mice (*n* = 5) were orthotopically implanted with U251 cells stably labeled with GFP-Luc into the right cerebrum and treated with vehicle, panobinostat (5 mg/kg/ip/5 days a week), LY500307 (5 mg/kg/oral/5 days a week) alone or combination and the survival of the mice recorded and plotted using Kaplan–Meier curve. (B) Mouse brains collected from the control and treatment groups were fixed in formalin and processed for IHC staining for Ki67. The number of Ki67-positive cells from 5 different images was counted and plotted as histogram. (C) Tumor sections were subjected to IHC staining for the detection of ERβ. Scale bar 100 µM. Data are represented as mean ± SEM. *P < .05; ***P < .001; ****P < .0001. Statistical differences were examined using log-rank test and 2-way ANOVA. ANOVA, analysis of variance; ERβ, estrogen receptor β; HDACi, histone deacetylase inhibitors; IHC, immunohistochemistry.

We next examined the efficacy of HDACi and ERβ agonist combination therapy on the survival of the tumor-bearing mice using in vivo orthotopic GBM model. U251 cells stably labeled with GFP-luciferase were implanted intracranially into SCID mice and treated with HDACi or LY500307 alone on in combination. We recorded the survival for tumor-bearing mice and analyzed the data using Kaplan–Meier curves. As shown in Figure 5A, combination therapy of HDACi and LY500307 significantly increased mice survival compared to monotherapy of HDACi or LY500307. Next, we studied the status of proliferation marker Ki67 in the tumors of control and treated groups using immunohistochemistry. The number of Ki67-positive proliferating cells was significantly lower in HDACi and LY500307 combination treated tumors compared to vehicle or monotherapy (Figure 5B). In addition, we examined the expression of ERβ protein in tumors immunohistochemically. As shown in Figure 5C, the expression of ERβ was significantly increased in HDACi treated tumors compared to vehicle treated tumors. Further, we also observed significant increase in ERβ expression in combination treatment group compared to monotherapy (Figure 5C). Altogether, these results suggest that HDACi treatment increases the ERβ expression and combination of HDACi and LY500307 improved the mice survival.

## Discussion

ERβ functions as a tumor suppressor in many cancers and its expression is reduced during cancer progression.^25–28^ Recent studies have shown that glial tumors express ERβ and this expression decreases during glioma progression.^15,25^ In this study, we tested the hypothesis that histone-mediated epigenetic changes contribute to alterations in ERβ expression and HDACi may upregulate ERβ expression. Our results showed that: (1) HDACi such as panobinostat and romidepsin have the potential to upregulate ERβ expression, (2) HDACi sensitizes GBM cells to ERβ agonist therapy in in vitro assays, (3) HDACi uniquely regulates expression of the ERβ1 isoform but not ERβ5 isoform, and (4) HDACi and ERβ agonist therapy enhances mice survival in orthotopic models. Collectively, these results suggest that HDACi and ERβ agonist combination may represent a novel therapy for GBM treatment.

Aberrant histone modifications are implicated in the development and progression of GBM.^29^ Specifically, HDACs, which cause histone deacetylation, and histone methyltransferases and demethylases that promote alteration in histone methylation are implicated GBM progression.^30^ Our results demonstrated that HDACi but not inhibitors of methyl transferases enhance expression of ERβ and suggest that alternations in histone acetylation may contribute to suppression of ERβ expression during GBM progression. Mechanistically, we demonstrated that HDACi increased expression of ERβ mRNA by promoting conducive changes in histone acetylation at the ERβ-0N promoter and ERβ target genes (MDA7 and NKG2E) promotors.

Gliomas are the deadliest tumors of central nervous system. Previously published studies have shown that HDAC 1, 2, and 3 are deregulated during GBM progression^31,32^ and HDACs negatively regulate estrogen receptors.^33,34^ Furthermore, the HDAC inhibitor, trichostatin A, sensitizes ERα-negative breast cancer cells to tamoxifen by upregulated expression and nuclear localization of ERβ.^35^ Our results are in agreement with these published studies and we conclude that HDACi has the potential to reactivate ERβ expression in GBM. Cell viability for cells treated with LY500307 and panobinostat combination is more affected in the U251 cell line than in the U87 cell line. These differences could be due to genetic differences in these 2 cells as U87 are positive for WTp53 while U251 are positive for MTp53. Interestingly, previous studies showed that ERβ interacts with MTp53 and promotes apoptosis.^36^ Since, ERβ functions as a tumor suppressor in GBM, these findings have implications in enhancing ERβ agonist therapies.

HDACs are categorized into 4 different classes: Class I (HDAC 1–3 and 8), Class IIA (HDAC 4–7 and 9) and IIB (HDAC 6 and 10), Class III (SIRT1–7), and Class IV (HDAC 11) based on their function and sequence homology to yeast proteins.^37^ HDACs play a critical role in cancer progression by inhibiting acetylation at promoters of genes involved in apoptosis, cell cycle, DNA damage, and angiogenesis.^38^ However, the mechanisms involved in regulating tumorigenesis by different classes of HDAC is not well understood. A recent study showed that HDAC 1, 2, 3, and 7 are significantly increased in grade III and IV glioma, while HDAC 4, 5, 6, 8, and 11 expressions are decreased in glioma patients.^39^ Panobinostat and romidepsin mediated increase in ERβ expression which was observed in our study may have been mediated by inhibiting class I HDAC isoforms. A limitation of our study is that we did not identify which specific HDAC enzyme contributes to the suppression of ERβ expression and future studies using CRISPR knockout (KO) of specific HDAC isoforms are needed to address this knowledge gap.

Recent studies, including ours, demonstrate that ERβ exhibits tumor suppressive functions in GBM and that high expression of ERβ was an independent favorable prognostic factor.^15,40–43^ Several studies demonstrated that gliomas express ERβ with low or weak expression of ERα. ERβ is highly expressed in low-grade astrocytoma and non-neoplastic brain tissues. In contrast, most of the high-grade tumors express low or decreased ERβ expression compared to low-grade gliomas, and this lower ERβ expression correlates with histological malignancy and poor survival of patients.^15,19,25,40^ Further, ERβ expression is downregulated or lost in several tumors including those of the breast, ovary, prostate, and colon.^15,28,44,45^ Our results suggest that HDAC-mediated epigenetic changes contribute to suppression of ERβ expression in GBM and HDACi such as panobinostat and romidepsin have the potential to enhance reexpression of ERβ.

The natural and synthetic agonists of ERβ exhibit antitumor activities in GBM and ERβ agonists reduce growth by decreasing the proliferation of tumor cells and by inducing apoptosis.^22,23^ Further, ERβ alters the chemosensitivity of cancer cells^23,46–48^ and ERβ agonists increase sensitivity of GBM cells to chemotherapeutic agents such as temozolomide and lomustine.^42,43^ Recent studies have shown that ERβ enhances chemotherapy response of GBM cells by downregulating DNA damage response pathways and chemotherapy induced activation of cell cycle arrest and apoptosis genes were attenuated in ERβKO cells.^23^ Panobinostat caused a delay in DNA damage repair after radiation treatment, inhibits migration and invasion of glioma cells and impairs tumor vascular formation.^49^ Romidepsin functions by downregulating the antiapoptotic protein Bcl-xL and upregulating p21 expression.^50^ In our studies, we also found that panobinostat and romidepsin promoted apoptosis and further enhanced ERβ agonist-mediated apoptosis. Interestingly, ERβ-KO attenuates the HDACi induced increase in p21 expression.

ERβ in humans is expressed as 5 different isoforms ERβ1, ERβ2, ERβ3, ERβ4, and ERβ5, resulting from alternative splicing of exon 8, which is the last coding exon, and these 5 isoforms add another layer of complexity in ERβ functions.^51^ Structural analysis revealed that ERβ1 is the only full-length functional isoform with the native ligand-binding domain.^51,52^ Off note, GBM cells express isoforms ERβ1 and ERβ5. Interestingly, expression of ERβ1 decreases with GBM progression with concomitant increase in the expression of ERβ5.^19^ Since, ERβ1 functions as tumor suppressor and ERβ5 functions as an oncogene, the observed alteration in the expression of ERβ isoform may reflect adaptation of selection pressures. Two promoters are shown to regulate expression of ERβ (0N, 0K) with 0N regulating ERβ1 and 0K regulating ERβ5.^52^ Published studies suggested that epigenetic but not genetic regulation of ERβ1 especially methylation adjacent to promoter 0N was a key regulatory event for ERβ1 silencing^53^ and that promoter 0K was normally unmethylated in cancer cells and tissues.^54^ Since we only observed changes in expression of ERβ1 with HDACi, we examined epigenetic modification at 0N promoter. Our results using multiple GBM cell lines indicated that HDACi regulate acetylation changes at 0N promoter. Since our previous study showed that ERβ5 is relatively expressed high in GBM compared to ERβ1, we speculate that there exist no epigenetic modifications of ERβ5 (0K) promoter to suppress its expression and hence no additional increase in expression of ERβ5 was observed with HDACi treatment as opposed to ERβ1 (0N promoter) which has relatively lower expression and is epigenetically regulated. It is possible that sequence/structural landscape of the 0N vs 0K promoter may contribute to differential regulation by HDACi.

In conclusion, our data have demonstrated that HDACi such as panobinostat and romidepsin have the potential to enhance the expression of tumor suppressor ERβ. Further, our studies implicate that upregulation of ERβ expression/functions by HDACi along with ERβ agonist is an attractive therapy for GBM.

## Notes

**Authorship Statement**. Designed the experiments and interpreted the results: G.R.S., R.K.V., R.R.T., S.M., and A.J.B. Conducted the experiments: U.P.P., S.V., Z.L., J.L., W.T., K.A.A., B.E., X.L., and G.R.S. Conducted xenograft studies: U.P.P, P.P.V., and G.R.S. Writing and editing of the manuscript: R.K.V., G.R.S., and U.P.P.
